# Chronic Obstructive Pulmonary Disease: An Analysis of Online Patient Testimony on Treatment Adherence

**DOI:** 10.3390/jcm14207324

**Published:** 2025-10-16

**Authors:** Laura Roldán-Tovar, Francisca Muñoz-Cobos, Francisca Leiva-Fernández

**Affiliations:** 1 Universidad de Málaga, Andalucía Tech, Faculty of Health Sciences, Arquitecto Francisco Peñalosa 3, Campanillas, 29071 Málaga, Spain; 2 Centro de Salud El Palo, Avda de la Estación Nº 2, 29018 Málaga, Spain; franciscamunozcobos@gmail.com; 3 Multiprofesional Teaching Unit of Community and Family Care, Primary Care District Málaga-Guadalhorce, Sevilla Street nº 23, 29009 Málaga, Spain; francisca.leiva.sspa@juntadeandalucia.es

**Keywords:** COPD, adherence, inhalers, medication beliefs

## Abstract

**Objectives**: The objective of this study was to explore the views expressed online by COPD patients regarding adherence to inhaled therapy. **Methods**: This study applied a qualitative, exploratory-interpretive design and an inductive methodology. Sources analyzed included COPD websites, patient forums, and social networks. Units of analysis were videos, stories, questions and answers, and conversation threads. Saturation criteria were applied. Applying a constant comparative methodology, analyses were conducted at textual (quotes, initial and focused coding, families) and conceptual (categories, networks, meta-network, provisional and final model) levels using ATLAS.ti 7.5. Reports were returned to patients. **Results**: There were 248 patients (51 men, 148 women, 49 unidentified) corresponding to 29 testimonies (6 narratives, 11 videos, 10 conversation threads, 2 questions collections). Adherence to inhalers is based on their perception of effectiveness to enable a normal life, and benefits should outweigh adverse effects. Adherence facilitators included mutual support between patients encouraging adherence and effective doctor-patient communication. Adherence barriers included (1) side effects; (2) mistaken beliefs about inhalers (habituation, attribution of non-existent side effects, fear of corticosteroids); (3) poor doctor-patient relationship (lack of listening, failure to consider patient’s preferences, communication iatrogenesis); (4) considering natural remedies as substitutes for treatment. **Conclusions**: Adherence to inhalers as reported in online testimony from COPD patients depends on the balance between efficacy and side effects. Adherence is influenced by peer support and doctor-patient communication. Doubts, erroneous beliefs, and iatrogenic effects of poor communication can hinder adherence.

## 1. Introduction

Chronic obstructive pulmonary disease (COPD) is a respiratory pathology characterized by chronic, generally progressive limitation of airflow [1]. According to the 2023 Annual Report of the Spanish National Health System [2], 22 cases of COPD were recorded in Spain per 1000 inhabitants (28.8 men and 15.4 women).

The chronic nature and symptoms of COPD influence patient quality of life at multiple levels: physical, due to the limitation of mobility, which hinders activities and impairs sleep quality [3]; social, due to decreased participation in activities and reduction in social relationships [4]; family, due to the need for physical support, difficulties fulfilling family roles, and a negative influence on sexual relationships; and psychological, due to the presence of anxiety and depression [5].

Mental models [6] are the individual’s representation of the world with which they interact; they are a result of perception, personal experience, and social interaction. Chronic diseases constitute a major component of the daily reality of people who experience them, and give rise to cognitive models based on one’s experience with symptoms and knowledge of the disease, as well as the context in which they develop.

The Leventhal Self-Regulation Model [7] is one of the most used methods to analyze these representations. It is based on the patient’s beliefs about their illness, which are categorized into five dimensions, identity, cause, timeline, consequences, and cure/control, the last of which includes the patient’s view of the treatment. This model has been used in research into the relationship between beliefs and control of respiratory diseases [8,9]. Horne et al. [10,11] designed a model of adherence to inhaled treatment called Necessity/Concerns, which classifies patients’ beliefs about treatments into two categories: perception of the need for treatment and concerns about potential adverse effects. This model has been used to investigate adherence in chronic diseases [10,11].

In terms of mental models of COPD, a Spanish study [12] found that only 40% of the general population evaluated knew of the disease, a relatively low percentage compared to other chronic diseases such as diabetes (98%) and asthma (97%). This lack of knowledge extends to patients affected by COPD, a previous qualitative study conducted in Spain highlighted the absence of a well-defined mental model of COPD, with the most confusing aspects being disease identity, diagnosis, the role of tobacco as the sole causative factor, exacerbations perceived as frightening but not recognized as part of the disease, and the presence of significant misconceptions regarding inhalers [13]. A qualitative study of patients who did not adhere to inhaled treatment [14] found that the majority did not have a clear definition of COPD, considered cure impossible, and felt that remaining stable was the best possible outcome. Most patients did not accept either their disease or the inhaled treatment. This lack of knowledge influences how patients experience symptoms and how they follow clinical recommendations [15].

Regarding types of non-adherence to inhaled therapy, three categories have been described: erratic (forgetfulness), deliberate or intentional, and unintentional (lack of knowledge). In patients with COPD, 47.8% of non-adherence is erratic, 34.1% intentional, and 31.2% unintentional [16]. Patient beliefs influence both intentional and unintentional non-adherence, as misconceptions arising from insufficient information may lead to treatment discontinuation either deliberately or unknowingly, with a substantial proportion susceptible to such beliefs.

Patients’ beliefs can be assessed through various sources of information, including the Internet. Internet use around disease is becoming increasingly important to patients, owing to the ease of access provided to information. A large percentage of people (74.9%) turn to digital media when seeking information on chronic diseases [17]. According to the CONOCEPOP II study, the following sources of information were used by COPD patients: media (35.5%), family and acquaintances (27.7%), and social networks/the internet (25.7%) [18].

COPD patients most commonly use the Internet to search for educational content, communicate with professionals, and seek support [19]. Internet use enables contact between groups of patients, providing feelings of belonging and social and emotional support [20].

There is growing evidence that online information affects treatment adherence in patients with chronic conditions. In a comparative analytical literature review [21] examining the impact of social media use by chronic patients on treatment adherence, the authors concluded that social media provided benefits such as obtaining support and accessing disease-related information. Although this review did not find direct evidence of social media influencing adherence to chronic treatments, the observed benefits suggest a likely emerging trend.

In Europe, previous studies have assessed the perspectives of patients with COPD via online interviews [22], rather than through analysis of patients’ spontaneous online posts.

Similar studies analyzing social media content exist, but they are limited to English-language testimonials. For example, in 2019, Apperson [23] in the USA examined the content of Facebook groups on COPD, including the communication strategies and participant engagement. Cook [24], also in 2019, performed a content analysis of social media platforms (Twitter, blogs, news websites, and forums) to investigate the perspectives of patients with COPD. The analysis included posts from both patients and caregivers, exclusively in English, and encompassed contributions from the USA, the UK, Canada, and Australia.

To date, no studies in Spain or in Spanish have investigated the perspectives of patients with COPD on their treatment online using the methodological approach of the present study, rendering this contribution a potentially significant advancement.

The aim of this study was to examine and understand the views expressed online by COPD patients regarding adherence to inhaled treatment.

## 2. Materials and Methods

### 2.1. Study Design

This study used a qualitative, exploratory interpretive design, applying inductive methodology, as per the Grounded Theory of Glaser and Strauss [25,26].

### 2.2. Participants and Units of Analysis

Participants were COPD patients who shared their experiences of the disease online, in Spanish, between 2010 and 2020.

**Figure 1:**
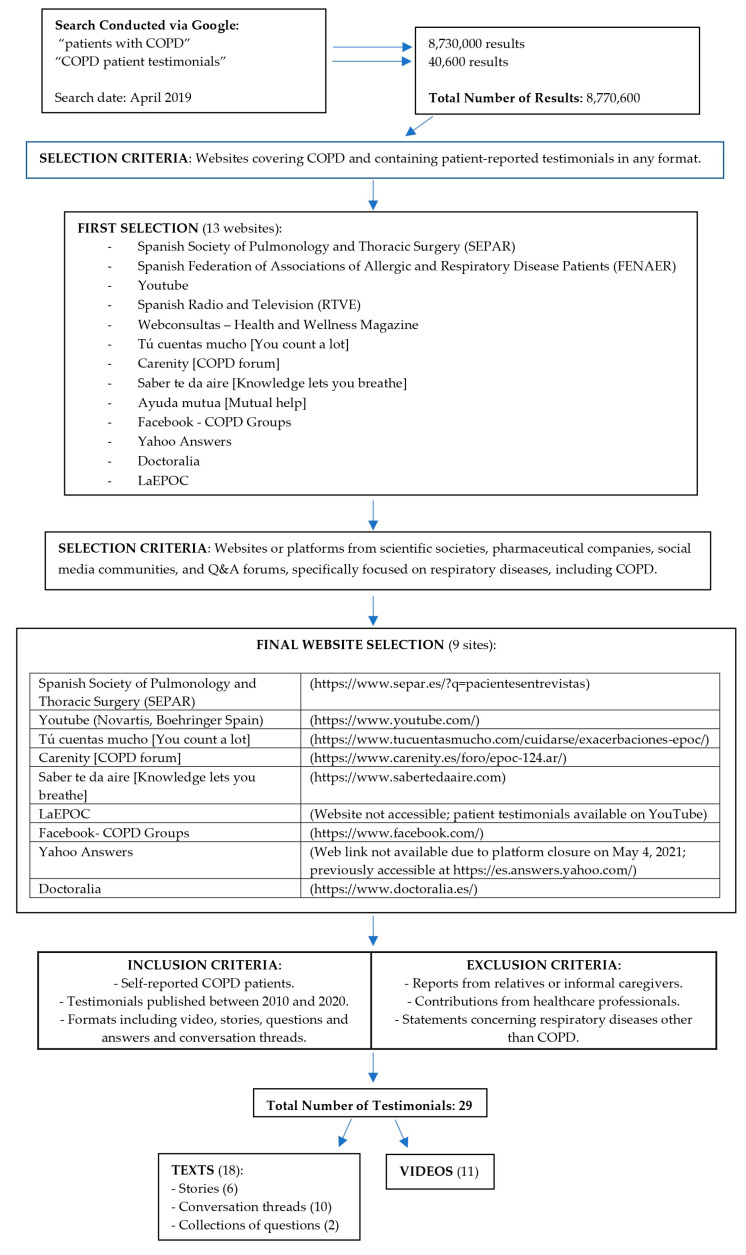
Procedure for Searching and Selecting Testimonials.

For the collection of testimonials, the initial selection of websites or platforms was conducted using the search terms “patients with COPD” and “COPD patient testimonials” in Spanish on Google. Websites providing patient testimonials or patient-to-patient interactions in any format were then selected. Figure 1 summarizes the search procedure and the selection criteria applied at each step.

Units of analysis were processed according to their respective modalities, following the procedures established by the software (ATLAS.ti 7.5, ATLAS.ti Scientific Software Development GmbH, Berlin, Germany):

Texts: Text-based testimonials were converted to rich text format and imported into ATLAS.ti for coding of patient expressions, and transcribed literally without modifications or spelling corrections. Language, structure, and content were analyzed, identifying themes, patterns, interactions, and underlying meanings. In conversation threads, the analysis also considered the use of emoticons, which added extra emotional nuance to the messages, as well as the “interactional climate” generated, reflecting metalinguistic aspects.

Videos: Videos were downloaded from YouTube and imported into ATLAS.ti, enabling simultaneous viewing and coding of patient expressions. Images, the patient prototype, statements made by and interactions between patients and other participants, the environment presented, and the key concepts of the video were analyzed. Notable nonverbal signals were documented.

### 2.3. Data Analysis

The analysis was conducted both at the textual and conceptual levels (Table 1).

All analytical procedures were carried out by two researchers with expertise in qualitative research and proficiency in ATLAS.ti. The analysis followed a cyclical process, conducted both concurrently and sequentially across the study phases.

### 2.4. Qualitative Techniques

The following qualitative techniques were used:

Memoing: Notes on comments, explanations or thoughts that arose during the analysis, allowing for thinking about the data, encouraging new ideas and relationships, facilitating the detection of errors and promoting cyclical decision-making in the research. The following types of memos used were comments, methodology-, theory-, and bibliography-related memos.

Triangulation: Allowed for comparison of data from multiple points of view. Different types of triangulation were used: data (video testimonies, text testimonies, conversation threads, questions and answers) and sources (open social networks and respiratory-specific websites).

Constant comparative method: Comparative analysis of the data to identify similarities and differences between components of the analysis (text quote-text quote, text quote-code, code-code, category-category, link-link, code-link-code, category-link-category, researcher-researcher).

### 2.5. Methodological Rigor

To ensure adequate representation, additional testimonials were included until analytical saturation was achieved (theoretical sampling). Sample diversity was ensured by selecting multiple data sources, including videos, stories, questions and answers, and conversation threads.

To validate the findings, member checking was performed. Upon completion of the analysis, a report summarizing the main conclusions in patient-comprehensible language (Section Feedback on Conclusions in Appendix A) was prepared and disseminated within a COPD patient forum. Participants were invited to indicate the extent to which they identified with the results. Facebook was selected for its accessibility and immediacy of interaction, specifically the official group “Foro-EPOC España—Asociación Española de Pacientes y Cuidadores de EPOC” [“Forum-COPD Spain—Spanish Association of Patients and Caregivers of COPD”], where prior consent for publication was obtained.

Original Facebook groups analyzed in the study were not used to avoid disrupting their normal functioning without moderator approval, and no responses were received from any of them.

## 3. Results

A total of 29 testimonies were included: 6 written narratives, 11 videos, 10 conversation threads, and 2 collections of questions (51 questions in total). The participating group consisted of 51 men, 148 women and 49 unidentified (anonymous or pseudonymous) participants.

The main reason expressed on the Internet for maintaining adherence to prescribed inhalers was the patient’s perception of efficacy and of benefits that outweighed any adverse effects.

Adherent patients acknowledged the benefit of inhalers (P27: I have changed inhalers 4 times in 20 years and have been using Breezhaler for 3 months, and I’m happy: one inhalation in the morning and that’s it, I can lead a normal life) and indicated that they were well adapted to inhaler use (P9: As for the treatment with inhalers, I am perfectly adapted, and, well, I have also perfectly adapted to how my disease has evolved).

Patients linked their adherence to their perception of benefits that outweighed the treatment’s side effects (P19: Hello Maria Jose, I also take several inhalers, some with Cortisone, and others with Albuterol. The latter cause tachycardia, and the cortisone ones cause sores in my mouth and tongue. Anyway, I’ll tell my view: what doesn’t give you wrinkles will make you fat…So I take everything I can to live as long as God wishes, in the best way possible).

Non-adherent patients indicated that they experienced no improvement with inhalers, even when recommended by their doctor (P23: What I don’t understand is why they treat me only with Ultibro: I don’t know, I have the impression that it doesn’t do anything for me, but he says it does).

They justified abandoning treatment due to the presence of side effects (P22: It was prescribed to me, but when I take it I cough a lot, and of course, you cough everything out), (P28: Ventolin makes me very nervous and I don’t use it).

Regarding factors that promote adherence to inhalers, the most important was support from other patients, specifically explicit advice to comply with the prescribed treatment. This included advice not to self-medicate and to consult a doctor about any doubts (P22: All medications are effective, but each body and illness responds in a different way. That is why one should never self-medicate. I never recommend medication to anyone, and I don’t think you should even ask anyone about it, other than the doctors who treat your illness), and revealed a delineation between offering emotional support and providing recommendations about treatments (P28: In this group we try to give emotional support, life advice, exercises, etc. But do not ask for or offer drug suggestions. That is up to our doctors. And herbal or dietary advice can be even worse…). Patients recognized the authority of the doctor in prescribing treatment (P28: Madam, don’t you have a doctor? I think that each case is unique and no one should tell you what to take, except your doctor), (P5: You should also stick to your medical treatment to the letter, take up to the limit provided every day, consult a doctor or your pulmonologist as soon as you experience a cough or shortness of breath), (P28: Well, whatever your doctor has prescribed. With this disease it is better not to take risks).

Various types of poor adherence were recognized, involving both omission and excess:

Discontinuous use and missed doses (P23: I think that without treatment, I only resort to medication when I feel bad… it is uncomfortable but it allows me to do everything at my own pace, I don’t use oxygen and I recently started using salbutamol 3 times a day, sometimes I forget).

Repeat dose (P29: When I inhale I cough and have difficulty obtaining the medication, so I repeat the inhalation several times. Should I stop taking it?).

Mis-use of rescue inhalers (P28: I have to nebulize myself with salbutamol up to 6 times a day, I always feel short of breath, and I feel very shaky from nebulizing so much).

Side effects were highlighted as factors that hindered adherence: Oropharyngeal fungal infections (P20: sometimes inhalers cause candida in the mouth), bad taste/loss of taste (P20: Before we had no taste because of tobacco, now it’s because of inhalers: such impotence), (P20: I always rinse afterwards but things taste bad to me), tachycardia/nervousness/tremors (P19: I used to use terbasmin (the blue one) and I had tachycardia and terrible tremors), voice problems (P28: I have been hoarse since they gave me symbicort), cough (P24: I use this spray… they switched it for Ultibro, but it didn’t work well for me, I coughed a lot when I inhaled it), sore throat (P28: My throat hurts a lot and I wash my mouth well after using the inhaler).

Patients had multiple doubts about inhalers (Table 2).

Misconceptions about inhalers were associated with poor adherence. The most relevant misconceptions were:

Habituation (fear of becoming dependent on inhalers) (P27: But I live in fear that one day my body will get used to the inhalers and I won’t be able to breathe. Every morning when I wake up I am reminded that I am short of breath, until I inhale my medication).

Fear of corticosteroids, and a belief that they are present in all devices (P26: All aerosols contain minimal levels of corticosteroids. And if you’re using them every day, just imagine).

Attribution of unrealistic side effects such as weight gain (P26: Being very fat is not good and I have gained a lot of weight since I started using these things), dry skin (P24: I took Ultibro and it is easy to take, but it dried out my skin a lot), hypertension (P26: WHAT’S THE NAME OF THE INHALER YOU USE? DOESN’T IT GIVE YOU TACHYCARDIA OR INCREASE YOUR BLOOD PRESSURE?), constipation and gas (P29: I use Ultibro daily and I notice constipation and gas).

Advice on the use of “natural” remedies, even as a replacement for medication, was common: steam (P26: I take medication for blood pressure and seretide only if I have noise in my chest, otherwise I don’t, but I steam before sleeping and I hardly use it, it’s mild-moderate for the moment), (P26: Seretide contains a corticosteroid and a bronchodilator. I’m going to stop using them: I’m going to manage with steam and oxygen only) and use of infusions, to which was attributed the same benefit as some medications, with the advantage of being “natural” (P28: Ginger and lemon infusions, you can make them or buy them already in bags, it works great for me).

Physician-patient communication was also a factor related to adherence. Patients indicated distrust in their doctor if they did not observe an effect of treatment, questioning the professional’s knowledge (P24: I have been doing this for a while with the inhaler, and at most I get 90, 93. I have the impression that my pulmonologist is ignoring me a little. I only take Ultibro 85/43 and I feel it’s not enough. Before I used to use another inhaler in the morning and another at night and I felt better, but they switched me to this one and no matter how many times I tell him that it makes me feel worse, he doesn’t listen to me). Patients disagreed with the recommended treatment and felt that their point of view was not considered (P24: When they insist on one medication, there’s nothing you can do. And the truth is that you know what’s good for you and what is not), (P24: Spiriva and Onbrez were what I took and I honestly felt better. But as I said, I told him and he said it was impossible, so that was that).

A lack of continuity of care when being seen by different professionals was considered a problem, as was the consequent lack of uniform criteria (P22: At the end of the month I have an appointment, let’s see what they tell me, I’m going to insist. I’m not always seen by the same person, and that’s a problem: everyone has a different opinion).

Patients demanded more psychological support from the professionals treating them, saying that they focused only on drugs and should also include psychological support (P25: Well, I’m trying to do something so that we can be protected. We don’t get any assistance for ourselves, I mean psychological. I would tell the doctors that not only does the drug cure you, but the brain goes in the other direction).

**Figure 2:**
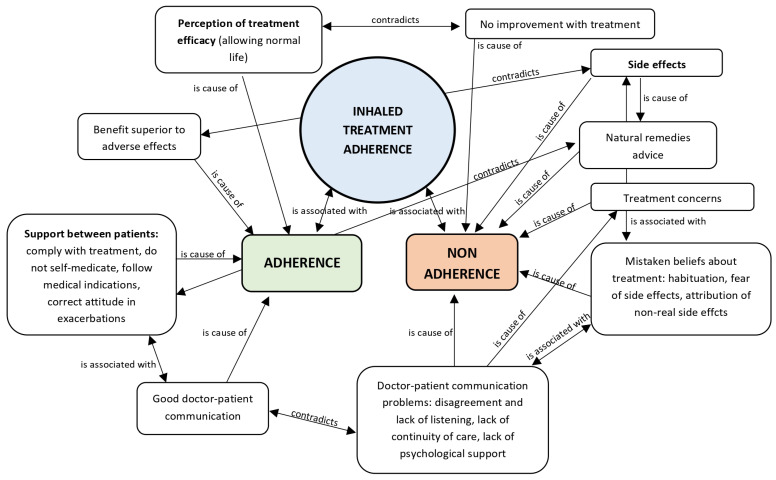
Relationships between factors associated with adherence to inhaled treatment.

The relationships between the different factors associated with adherence to inhaled therapy that lead to the reported results are shown in Figure 2.

## 4. Discussion

The key finding regarding adherence to inhalers in online patient testimony was the balance between perceived benefit and adverse effects. This finding is consistent with Horne’s Necessity-Concern framework [10]: adherence behaviors are based on the balance between the perception of the need for treatment using inhalers and concerns about their use, including fear of side effects (real or imagined) and erroneous beliefs. In our study, “necessity” was linked to perception of an effect, which in practice meant the ability to lead a normal life. If the balance between functional benefit and fear of adverse effects was negative, the treatment was perceived as of little use, or even harmful, justifying non-adherence behaviors as described by Horne [10,11]. Hometowska et al. [27] examined adherence to inhaled therapy among Polish patients with chronic obstructive pulmonary disease and reported findings consistent with both our results and Horne’s model: stronger beliefs in the necessity of medication were positively correlated with higher adherence across all dimensions, whereas greater concerns regarding potential medication-related harm were negatively correlated with adherence to inhaled treatment. This explanatory model of non-adherence has likewise been observed in other respiratory diseases, such as asthma, where in a non-experimental, cross-sectional, correlational study conducted in Mexico, patients with asthma completed the Revised Illness Perception Questionnaire, the Beliefs about Medicines Questionnaire, the Medication Adherence Report Scale–Asthma, and the Asthma Control Test, with findings indicating that patients’ perceptions of treatment were found to influence their illness perceptions, adherence behaviors, and overall asthma control [28]. Other studies, such as that of Suárez-Argüello et al. [29] in a sample of Mexican patients with hypertension and that of Pagès-Puigdemont et al. [30] in a literature review of patients with chronic diseases, have also reported that adherence can be negatively influenced by beliefs about the disease and the treatment, unrealistic expectations about the effect of treatment, and fears that it may even be harmful.

Patients on the Internet expressed multiple doubts regarding inhalers (effect, side effects, dosage regimens, incompatibilities, etc.). This lack of clarity, manifested as doubts, has also been reported by Kuipers et al. in a qualitative study conducted through telephone interviews in the Netherlands, in which patients with COPD expressed doubts about the clinical indication, treatment duration, and potential side effects of inhalers [31]. In a cross-sectional study of patients with COPD, Duarte de Araújo et al. [32] found that doubts or confusion, fear of side effects, and concerns regarding long-term toxicity or tolerance—reflecting a broader lack of information about inhalers—contributed significantly to poor adherence.

Patients also expressed misconceptions regarding inhalers, consistent with findings from other qualitative studies that have reported the absence of a well-defined mental model of COPD and the presence of erroneous beliefs about treatment [33]. Among the misconceptions identified were: the fear of corticosteroids and the belief that corticosteroids were present in all inhalers. This negative perception of oral steroids and the lack of confidence in them has previously been reported in patients with COPD by Román-Rodríguez et al. in an international qualitative study [34]. Patients also mentioned concerns about becoming accustomed to or dependent on inhalers. Similar beliefs have been reported by Chan et al. [11] and Amin et al. [35] in patients with asthma, who expressed the same worries about long-term effects of inhaled treatment and becoming overly dependent on their inhalers. Additionally, patients attributed unlikely side effects to inhalers, including weight gain, dry skin, hypertension, constipation, and flatulence. These erroneous assumptions about inhaler-related side effects have also been reported in other studies, in which patients cited weight gain, muscle pain, dizziness, elevated blood pressure, and skin-related effects [35,36].

Patients highlighted problems with doctor-patient communication: lack of confidence, dissatisfaction with treatment and failure on the part of the doctor to modify their approach based on the patient’s point of view. The lack of a relational strategy in prescribing, leading to patient mistrust, non-adherence to treatment, and poor disease control, could be considered an iatrogenic effect of poor communication. Australian patients with COPD in a qualitative study interviewed by Madawala et al. [37] revealed that a lack of confidence in their general practitioner, together with the perception that their physician had limited knowledge of and interest in the disease (a recurrent concern among participants), led them to communicate less with their doctor and, consequently, to be inadequately informed and treated. Similarly, unintentional non-adherence to inhalers has been attributed to factors such as inadequate communication with healthcare providers, insufficient understanding of the necessity or proper instructions for use, and forgetfulness [38].

Other studies of chronic diseases have identified an inadequate doctor-patient relationship was a risk factor for poor treatment adherence, as evidenced in the case–control study conducted by Mujica in hypertensive patients [39], with physician empathy [40] and patient knowledge about COPD [41] identified as factors promoting adherence. Sigurgeirsdottir et al., in a phenomenological study using in-depth interviews, evaluated the needs of patients with COPD and also identified the need for good healthcare and found that patients criticized a lack of interest (on the part of the doctor) and of continuity of care [42].

Another issue detected by patients was the provision of care by different professionals, and a consequent lack of uniformity in medical criteria. Lasmarías-Ugarte et al. [43], in a Spanish qualitative study, reported that treatment adherence in patients with COPD is influenced by the trust and support they receive from healthcare professionals, and concluded that person-centered care is key to improving adherence.

Patients indicated the need for more psychological support from professionals, who tended to focus on pharmacotherapy while ignoring the associated psychological burden. In agreement with this finding, Russell et al., in a systematic review of qualitative research [44], concluded that patients’ psychosocial needs should be prioritized along with medication and exacerbation management, and patients’ personal beliefs about COPD should be reviewed periodically.

There may be a relationship between lack of knowledge about COPD, a lack of communication with the doctor, and use of the Internet to search for answers. A European study [45] found that three-quarters of patients used the Internet to search for information about COPD and one-third to exchange experiences about their disease; notably, according to Apperson et al. [23], medication management was the most frequently discussed topic in Facebook groups focused on COPD.

Patients acknowledged that the Internet is not a suitable forum to pose questions about their medication, consistent with González-Barberá et al. [46], who reported in a qualitative Spanish focus group study that patients did not trust online forums as sources of information for treatment-related decision-making, although they concluded that the Internet was a valid source of information and provided emotional support. Similarly, in a qualitative study, Ali et al. [47] reported that patients with COPD found support through the knowledge and experiences shared with other patients.

A fundamental strength of this study is its analysis of online content to explore the perspective of COPD patients, and its use of a qualitative methodology to explore patient opinions, experiences and beliefs expressed via a wide range of formats. The specific characteristics of the Internet (anonymity, online interpersonal contact) allow for more open expression of personal opinions than may occur in face-to-face interviews [48].

Regarding limitations, we sought to avoid subjectivity by following the recommendations of Auerbach and Silverstein [49], as follows: (1) Transparency (describing all the steps taken); (2) Communicability (clearly expressing all information obtained); and (3) Coherence (continuous return to the testimonies in all phases of the analysis; analysis by two researchers; use of triangulation between different sources, and application of a constant comparative method [50]).

There are biases inherent to the use of the Internet as a source of information [51]:

(1) Patient self-selection: Patients who use the Internet do not always match the typical profile of individuals with COPD. According to a cross-sectional study conducted with patients attending their healthcare center in Spain [52], Internet use was associated with younger age (mean 54 years) and higher educational level (completion of secondary or higher education). These characteristics differ from the typical profile of patients with COPD in Spain, where the mean age is higher (66.5 ± 10.9 years) and the proportion of patients with medium-to-high educational level decreases [53]. This approach could overlook the perspectives of patients who are older, less educated, or less likely to use the Internet. Although younger individuals are generally more frequent Internet users, age may not be as strong a determinant of online representation in certain countries. For instance, a Swedish study with a mean participant age of 72.5 years reported that over 90% had Internet access and demonstrated high proficiency in its use [54]. In Spain, according to the INE [55], 80.1% of individuals aged 65–74 years used the Internet in the past three months in 2023, representing an increase of 6.8 percentage points from 2021 (73.3%) [56]. Among those aged 75 years and older, Internet use also increased: the most recent INE data from 2022 [57] indicate that 35.9% of this population accessed the Internet in the past three months, up 8 points from 2020 (27.9%) [58]. Given the demographic profile of patients with COPD in our country (mean age 66.5 ± 10.9 years) [53], they fall within a moderate-to-low but steadily increasing range of Internet use. This should be taken into account when extrapolating the findings of our study beyond Spanish-speaking COPD patients who routinely use the Internet.

(2) Bias related to the choice of sources and bias related to the origin of the information (why is this content accessible while other content is not?; why and by whom were these testimonies uploaded to the Internet?; are all testimonies complete or is some relevant data omitted?). We sought to control for the above by using various sources and different types of testimony until reaching saturation.

Other potential biases include:

Generalization to Patients Speaking Other Languages: This study may be subject to bias as only Spanish-language patient testimonies were included; however, based on the literature, Spanish patients with COPD share characteristics with patients from other European countries, supporting the potential generalizability of our findings to Internet-using COPD patients in other languages.

- Patient profile: Compared with COPD patients from other countries, Spanish COPD patients are predominantly male (54.1% vs. 41.6% female), with a mean age of 66.5 ± 10.9 years, a mean BMI of 27.4 ± 4.8, and 73% with a history of tobacco use (30.9% current smokers, 42.1% former smokers). They also tend to have a lower educational level and more comorbidities compared to non-COPD controls [53]. This profile is consistent with COPD populations in countries such as Greece [59], Austria, Bulgaria, Croatia, Czech Republic, Hungary, Latvia, Poland, Russia, Serbia, Slovakia, and Slovenia, as reported in the POPE study [60].
- Adherence levels: Poor adherence to inhaled therapy is a widespread phenomenon. In Spain, only 57% of patients were reported to use inhalers correctly [61], consistent with findings from other countries and cultural contexts, including China [62], Greece [63], and Latin America [64].
- Adherence-related factors: Patient beliefs have also been associated with adherence. In a Chinese study, a strong correlation was observed between disease perception and inhaler adherence [65]. More broadly, a systematic review of English-language literature from high-income countries [66] indicated that adherence is influenced by patients’ beliefs and experiences regarding medications, satisfaction with treatment efficacy, concerns about side effects, personal circumstances, habits, health status, and relationships with healthcare professionals. Therefore, determinants of inhaled therapy adherence are multiple but do not differ significantly across countries.
- Internet use: Internet use and social media engagement in Spain are comparable to the European average. According to Eurostat, in 2024, 58.16% of Europeans sought health information online, with Spain above the average at 69.63% [67]. Participation in social media (creating profiles, posting messages, and other contributions on platforms such as Facebook and Twitter) was 64.82% across Europe, with Spain at 64.70% [68]. Although data on Internet use specifically among Spanish COPD patients are limited, studies from other European countries indicate that COPD patients actively engage in the digital society, using the Internet to seek information and share experiences with peers, as reported in studies from Sweden [54], Germany, and Switzerland [45].

In summary, considering the similarity of the Spanish COPD patient profile to that of other European countries, our findings could reasonably be extended to Internet-using COPD patients in other languages. Nevertheless, future research could consider incorporating multilingual data to further evaluate and extend these findings.

The use of the Internet as an information source provides insights into patient experiences and opinions, but concerns regarding its reliability persist. This study is novel, representing a first approach using social media content analysis to explore the perspectives of Spanish-speaking patients with COPD. Although the findings were validated by patients from the social network through feedback on the report, future studies are needed to determine whether these results can be replicated.

This study does not provide demographic characteristics of the patients due to the impossibility of collecting such data while maintaining Internet anonymity. Nevertheless, as previously noted, anonymity also offers advantages, such as facilitating the spontaneous expression of experiences and opinions.

Our data were not stratified by sex, and the unbalanced representation between men and women (51 and 148, respectively) could potentially influence perspectives on adherence. However, as this is a qualitative study using an inductive methodology, no sex-related influence was detected in patient testimonials. Regarding the use of the Internet for health-related purposes, women are predominantly represented. In a comparative study across European countries on Internet use for health information, the proportion of women (77%) was higher than that of men (65.4%), and social media platforms were more frequently consulted by women [69]. These findings are consistent with a systematic review, which also reported that women are more likely than men to seek health information online [70]. Similarly, in a descriptive study in the United States involving patients with chronic conditions, women used the Internet more frequently (56.7%) compared to men (43.3%). This may be explained by the tendency of women with chronic illnesses to adopt a more proactive approach to managing their health [71]. In our study, a similar pattern of higher female representation was observed. Moreover, previous scientific literature has not established a clear relationship between sex and adherence. In a study conducted across primary care centers throughout Spain [16], adherence to inhalers was measured using the TAI questionnaire, showing adherence rates of 49.7% in men and 45.7% in women, with a weak association between sex and adherence. Duarte de Araujo [32] conducted a study in Portugal and reported no statistically significant relationship between sex and inhaler adherence. In fact, numerous studies assessing inhaler adherence did not stratify results by sex either [62,63].

A potential limitation of this study is the possibility that dissatisfied patients may be more likely to use social media to express complaints, and we cannot rule out their overrepresentation. This phenomenon has been observed in extreme adverse situations, such as during a pandemic [72], or when patients are asked to provide feedback on a service or healthcare professional [73]. In our study, however, this bias was not evident, as the collected testimonies reflect experiences with the disease that include both negative and positive aspects. Notably, the predominant message conveyed is one of encouragement and support for other patients, rather than expressions of dissatisfaction.

Recommendations for Clinical Practice: Our findings provide useful insights on practical means of improving adherence to inhaled treatment. They suggest that it may be useful to explain to patients the efficacy and adverse effects of inhalers, and the benefit of treatment and the timeframe in which this will be evident, and to monitor for potential adverse effects. This type of individualized assessment would help ensure that treatment efficacy, measured in terms of functional improvement, is sufficiently evident such that the patient can perceive the beneficial effects of the inhaler. Explaining treatment guidelines and the correct use of rescue medication can improve adherence. Knowledge of patients’ erroneous beliefs and specific doubts about inhalers, which could lead to abandonment of treatment, can help healthcare professionals anticipate lack of adherence and intervene to resolve patients’ doubts. Agreement on treatment is a key need reported by patients, who want their doctors to listen to them and involve them in the decision-making process. This in turn will facilitate treatment adherence.

## 5. Conclusions

In conclusion, online testimony from COPD patients indicates that adherence to inhalers is dependent on the perceived balance of benefits to adverse effects. Perception of the treatment as effective (capacity to perform activities of daily living) promotes adherence, while lack of improvement, adverse effects (real or perceived) and problems with doctor-patient communication hinder adherence.

## Figures and Tables

**Table 1:** Analysis process.

Textual Level	Conceptual Level
1. Preparation of primary data: Transformation to rich text, to facilitate the exchange of documents between different computing platforms. 2. Reading and rereading: Allowed for a global understanding of the transcribed testimony and the fundamental ideas expressed, and familiarization with the language used by the participants. 3. Segmentation of the text into quotes: Text fragments (quotes) relevant to the research objective were identified. 4. Initial coding: A code was applied to each selected text or video quote. Comments were added to the codes to clarify their meaning. The dictionary of the Royal Spanish Language Academy was used in case of doubts about terminology. 5. Focused coding: Review of codes to correct errors, recode, or merge multiple codes with the same meaning. This was carried out (1) in parallel with the first round of coding, when new codes emerged, and previous codes had to be revised; and (2) upon completion. 6. Families: Classification and ordering of codes according to common elements.	7. Networks: Visual diagrams based on families made up of nodes (codes considered categories with specific meaning due to their position and relationships within the network) and relationships between nodes (links: “is associated with”, “contradicts”, “is a property of”, “is part of”, “is a cause of”, “is a”). Characteristics of the networks: Degree (number of links from each node) and Order (number of nodes in the network). Network analysis: high-degree nodes (higher-level explanatory codes): higher Density (number of quotes referred to) and Foundation (number of relationships with other codes). These nodes were considered central categories of the network. Meta-network analysis: simultaneous analysis of the main nodes of each network. 8. Provisional and Final model.

**Table 2:** Questions about inhalers.

Doubts	Sample Quotes
About its effect	Uncertainty about possible improvement (P29: For my COPD treatment I was prescribed inhalers, Foster Nexthaler and Eklira Genuair, will I get better?) Confusion between lack of effect and worsening of disease (P22: I take it, but I honestly think I was better when I was taking Spiriva and Onbrez. I feel like I’m choking, I don’t know if it’s because I’m worse or because it’s not doing me any good)
About side effects	(P20: Good evening group, a question, do inhalers give you fungus? I have a fungus in my mouth and my entire throat)
About usage time	(P29: For how long is it advisable to take Enurev to improve COPD?)
About usage guidelines	(P29: I have COPD and use Atrovent. Spiriva. Onbrez. Is it necessary to nebulize with Atrovent every day? Can I use Spiriva and Onbreze every day? How long should I leave between one inhaler and the other?)
On incompatibilities between inhalers	(P29: I have been admitted for an exacerbation of COPD with emphysema. When I was discharged, as a continuing treatment, they prescribed me Ultibro, which I was already taking, a single inhalation in the morning, and Alvesco, one in the morning and one at night. Are the two compatible?)
On the use of rescue treatment and its appropriateness	(P29: I am a 42-year-old woman with moderate COPD but still without symptoms. I play sports (basketball, gym) and since I am so young and have moderate COPD, they told me that I could be a patient with freefalling FEV…I have only been prescribed Ventolin as a rescue treatment. What do you think about treatment and being a “free falling” patient”?)
On treatment in exacerbations	(P29: I just dosed myself 3 h ago with 1 ventolin and 2 atrovent inhalers, and I have nebulized with physiological saline solution but my breathing difficulty continues. I suffer from COPD and have just been diagnosed with acute bronchitis. Will taking prednisone help me? What should I do?)

## Data Availability

The original contributions presented in this study are included in the article. Further inquiries can be directed to the corresponding author.

## Abbreviations

The following abbreviations are used in this manuscript:

COPD	Chronic Obstructive Pulmonary Disease
TAI	Test of Adherence to Inhalers
INE	National Institute of Statistics

## Appendix A. Feedback on Conclusions

Hello everyone,

We have conducted a study on how patients with COPD perceive inhaler treatment, based on their testimonies on the Internet.

We would like to share the preliminary conclusions of the study with COPD patients so that you can review them and give us your opinion, specifically whether you feel more or less reflected in the ideas presented. Your feedback will be taken into account in the final conclusions. Personal data confidentiality is guaranteed; no names or identifying information of participants will appear.

You can comment here if you feel identified with these ideas or not, or alternatively, you can send your feedback to our email: estudioepocinternet@gmail.com

Thanks go to the Spanish COPD Association for their consent and support in this project.

We would greatly appreciate your help.

The main ideas extracted from the study are:

The main use of the Internet for patients with COPD is support among patients. Contacting other people with COPD online promotes a sense of belonging to a group, as they feel understood and listened to by their peers.

Most patients are satisfied with the support and care received from healthcare professionals, but they also describe negative experiences, such as not always being seen by the same doctor and communication problems with professionals when patients’ opinions about their treatment are not taken into account.

Regarding inhaler treatment, correct and sustained use depends on the balance between checking that they are effective (allowing a normal daily life) and whether they cause unpleasant side effects for the users. Patients have many doubts about how they should be used, their expected effect, dosage, whether they can be combined with other treatments, and potential side effects. Beliefs also appear, such as fear of corticosteroid effects, worry about “becoming dependent” on inhalers, and the perception that they produce more negative effects than have actually been demonstrated with their use.

Warm greetings and best wishes for your health.
